# Effectiveness of caregiver-mediated intervention: a pilot study for children with neurodevelopmental disorders

**DOI:** 10.1017/S1463423622000524

**Published:** 2022-10-14

**Authors:** Dongmei Ge, Hua Wei, Yue Wang, Yan Li, Jinmei Luo, Xiao Liu, Yan Hu, Li Chen, Qian Cheng, Tingyu Li, Ying Dai

**Affiliations:** Department of Child Health Care, Children’s Hospital of Chongqing Medical University; Ministry of Education Key Laboratory of Child Development and Disorders; National Clinical Research Center for Child Health and Disorders; China International Science and Technology Cooperation Base of Child Development and Critical Disorders; Chongqing Key Laboratory of Child Health and Nutrition, Chongqing 400014, China

**Keywords:** Autism, caregiver-mediated intervention, neurodevelopmental disorders, primary health care

## Abstract

**Objectives:**

Caregiver-mediated intervention (CMI), based on parent skills training, is a family-mediated intervention model for children with neurodevelopmental disorders, in particular autism spectrum disorder. This study aimed to evaluate the effectiveness of CMI.

**Methods:**

Thirty-three children (aged 22–69 months from our department) and their caregivers participated in a two-week training course of ten 90-minute lessons. Caregivers were encouraged to try their best to apply intervention skills in both home routines and play routines to encourage the development of cognition, motion, social adaptability, and behavior of children. Demographic information, video-recorded data, and diagnostic scales were collected at two key time points: baseline and post-training (PT – within six months).

**Results:**

Three aspects were assessed – primary variables, secondary variables, and correlation analyses. Results showed an improvement in PT in (1) Adult/Child Interaction Fidelity Rating (*P* < 0.01) and (2) adaptability of Gesell Developmental Scale and stereotyped behaviors and limited interests of Autism Diagnostic Observation Schedule (*P* < 0.05, *P* < 0.01). Moreover, a negative correlation occurred between caregiver skill improvement and parent education (*P* < 0.05), but without correlations with other demographics.

**Conclusions:**

As an efficacious family intervention for both children and their caregivers, CMI is worth being generalized widely.

## Introduction

Neurodevelopmental disorders (NDDs) in children are a class of chronic developmental cerebral dysfunction caused by complex genetic and environmental factors, which affect the cerebrum functional areas of cognition, motion, social adaptability, and behavior, such as autism spectrum disorders (ASD), global developmental delay (GDD), intellectual disability (ID), and attention deficit hyperactivity disorder (ADHD) (Zinkstok and Buitelaar, 2014, Peng and Yin, 2018). With increasing morbidity levels year on year, it has been reported that the morbidity of NDDs in Taiwan rose from <1.3% in the early 1990s to nearly 3% in 2016 (Wang, 2016). As the representative diseases, ASD and GDD have no specific therapeutic characteristics. The need for lifelong intervention is essential, meaning heavy burdens for everyone involved, which requires a long intervention period and the joint effort of hospitals, families, educational institutions, and communities. Because children with NDDs have close contact with family caregivers, day and night, family-mediated intervention models are important. Compared with multitudinous intervention of models, targets, and assessments in ASD in the rest of the world, systematic and scientific family-mediated intervention models are still lacking in China (Ge and Dai, 2019). China has a large population of children with ASD, which means intervention resources are relatively inadequate.

Under the circumstances, family-mediated intervention models, as low-intensity interventions (Weitlauf and McPheeters *et al.* 2014), are relatively low in cost and adaptable for the clinic or home and for groups or individuals (Lord *et al.* 2018). In 2008, the World Health Organization (WHO) launched the Mental Health Gap Action Program (mhGAP) to facilitate the achievement of targets for the Comprehensive Mental Health Action Plan 2013–2020. The original intention of mhGAP was to help people with mental, neurological, and substance use (MNS) disorders by reinforcing the commitment of governments, international organizations, and other stakeholders to increase the level of financial and human resources, particularly by improving the coverage of key interventions in low- and middle-income countries. In 2010, mhGAP-Intervention Guide (mhGAP-IG), version 1.0, was released and translated into several official languages of the United Nations to provide evidence-based guidance for all WHO regions. In 2015, mhGAP-IG, version 2.0, was released. In the guidance, parent skills training (PST) (Rahman, 2017) was recommended as a key intervention strategy for caregivers of children aged 2–9 years with developmental disorders/delays to improve the caregiver/child relationship, promote the child’s functioning and developmental outcomes, and sustain the caregiver’s role and function. Research in Pakistan (Rahman, 2017), as one of the three randomized controlled trials (RCTs), follows a cascade model. First, the master trainers will train ten trainers through 10 days of classroom session. The trainers then provide guidance for “champion” family volunteers who are caregivers of children with developmental disorders/delays for a duration of at least six months, and lastly, the volunteers deliver the training consisting of nine weekly sessions to four or five families in their villages.

In view of the current medical and health situation in China, the number of patients with NDDs requiring intervention is massive, and at the same time, access to family intervention through the community is still not available. This study was designed to analyze the intervention effectiveness of caregiver-mediated intervention (CMI) based on PST in order to provide some evidence for a domestic family intervention model.

## Methods

### Inclusion criteria

All participants met the diagnostic criteria for ASD or GDD in the Diagnostic and Statistical Manual of Mental Disorders, 5th Edition (DSM-5), as well as a clinical decision by a physician with complete related diagnostic test scale results before participating in training. Participants had no severe acute or chronic diseases and were of age between 18 and 72 months. The participants had no previous systematic training. The caregivers signed the informed consent form of the course and video recording, uploading at least two videos at two key time-points: baseline (BL) and post-training (PT; within six months), presenting the interaction about home routines or play routines, with about 10 min every video.

### Participants and ethical considerations

Thirty-three children and their caregivers who participated in the CMI course in the Department of Child Health Care of our hospital, from September 2018 to June 2019, met the inclusion criteria and were enrolled. Participants were 30 boys and three girls. The ages ranged from 22 to 69 months, and the mean age was 39.61 ± 13.12 months. The Institutional Research Ethics Boards approved the study and caregivers provided informed consent prior to participation. Thirty-one children were confirmed with ASD and two with GDD. Caregivers filled in information when participating in CMI. Table 1 shows the family characteristics.

**Table 1.** Family characteristics of participants including residence, main caregiver, family structure, parental education, and annual household income

Variable	Classify	Count (NS 33)	Percentage (%)
Residence	City	33	100.00
	Country	0	0.00
Number of children in family	One	33	100.00
	More than one	0	0.00
Main caregiver	Father/mother	11	33.33
	Parents	13	39.39
	Grandparents	9	27.27
Family structure	A couple and their child	10	30.30
	Three generations under one roof	21	63.64
	Four generations under one roof	2	6.06
Father education	Primary or middle school	3	9.09
	High or technical secondary school	5	15.15
	College or junior college	24	72.73
	Master degree and above	1	3.03
Mother education	Primary or middle school	3	9.09
	High or technical secondary school	4	12.12
	College or junior college	25	75.76
	Master degree and above	1	3.03
Annual household income	<100,000RMB	17	51.52
	110,000–150,000 RMB	7	21.21
	160,000–200,000 RMB	4	12.12
	>200 000 RMB	5	15.15

### Procedure

CMI is based on the theory of applied behavior analysis (ABA) and is applicable mainly for children aged two to six years with NDDs and caregivers who had not received systematic training previously. The CMI consisted of ten 90-minute sessions, two-week teaching cycles, and it was carried out by qualified trainers from our department who were trained and supervised by the master trainer of PST through the standard training videos. Parent-theory teaching and parent–child interaction proceed simultaneously. One caregiver attended to parent-theory teaching alone, including (1) introduction of CMI and encouraging children to participate; (2) appropriate home routines and play routines; (3) learning the way of communication and prompting interaction; (4) prompting communication and developing language; (5) regulating the child’s emotions; (6) teaching alternatives to challenging behavior; (7) teaching children new skills; (8) self-care and adjustment of caregivers; and (9 and 10) practices and comments. At the same time, another caregiver with the child participated in group courses divided into music, games, snacks, and exercise modules. At the end of the two-week course, caregivers were encouraged to try their best to apply the theory and intervention skills in the daily life, including home routines and play routines, to prompt the development of cognition, motion, social adaptability, and behavior. Demographic information was collected, and video data and diagnostic scales were recorded at two key time points at least: baseline (BL); post-training (PT; within six months).

### Video-coded scheme and assessment measures

Data recorded in the videos before and after intervention were coded as the primary outcome variables. The caregivers were encouraged to record ten-minute videos of caregiver–child interaction in home routines or play routines to evaluate if the caregivers demonstrated appropriate use of techniques including (1) environmental sets (i.e., the caregiver sets up the environment and moves to be face to face to promote engagement in play or home routines); (2) offering choices (i.e., the caregivers offers choices for the activity, follows the child’s appropriate choices and interests, and comments on the child’s focus of attention and actions); (3) building and sustaining routines; (4) pauses (i.e., the caregivers pauses and allows space for the child to communicate); (5) responding (i.e., the caregivers notices the child’s communication and responds promptly by expanding it by imitation, expansion, and modeling of words and gestures); (6) matching (i.e., the caregiver provides communication models that match the child’s communication goals, including gestures and spoken language); (7) requesting (i.e., the caregiver sets up the environment to create opportunities for communication to request); (8) sharing (i.e., the caregiver sets up the environment to create opportunities for communication); (9) supporting and regulating (i.e., the caregiver supports the child’s engagement and regulation throughout the interaction); (10) different degrees of assistance (i.e., the caregiver promotes child’s learning of new skills by using instructional strategies into developmentally appropriate small steps and teaching one step at a time, constructing and applying visual supports as needed, giving the lowest level of help needed for the child to be successful, and reducing the level of help in a timely way); and (11) challenging behaviors (i.e., if challenging behavior occurs, the adult responds by demonstrating use of appropriate strategies to reduce the behavior and support the child’s regulation based on the function of the behavior). Based on those 11 strategies, the question (Q) 1 to 10 of Adult/Child Interaction Fidelity Rating (A/CIFR) was matched with the strategy from 1 to 10 above, and the strategy (11) is set as an additional question marked “×”. There were five levels for every question: 0 – the skill/strategy is not demonstrated or inappropriately applied when opportunities are present; 1 – the strategy is used somewhat appropriately at times; 2 – implementation of the strategy is mixed – about 50% of the time the strategy is correct; 3 – appropriate and accurate implementation of the strategy occurs up to 80% of the time; and 4 – strategies are applied appropriately in 80%–100% of opportunities.

In the preliminary stage, the writer watched the reference videos of CMI and learned the corresponding scoring rubrics of A/CIFR when scored by experts. The writer and another author trained by experts evaluated 12 videos of four children with NDDs (total time: 137 min 58 s) synchronously, and the consistency test was 99.24%.

### Standardized measures

Baby-junior high school students’ ability of social life scale (S-M) (Zhang *et al.* 1995, Huang *et al.* 2018), based on S-M Social Life Ability Examination established by Japanese Institute of Psychological Adaptation, was revised by professors of Beijing Medical University and Chinese Psychological Society, mainly for Chinese children with mental retardation aged 0–14 years. The S-M was used to evaluate six aspects of ability, including self-help, locomotion, occupation, communication, socialization, and self-direction, as important as Gesell Developmental Scales (GDS) for the diagnosis of GDD.

GDS (Liu *et al.* 2018) were compiled by A Gesell, an American psychologist, and revised twice in 1941 and 1980. It focused on five areas of ability – gross motor, fine motor, language, adaptability, and individual-social behavior, mainly for children with GDD aged from four weeks to six years. The result of the GDS shows developmental quotient (DQ) instead of intelligence quotient (IQ). It is used widely in China, not only as the golden standard for the diagnosis of GDD but also as an auxiliary tool for the diagnosis of ASD. One study (Liu *et al.* 2019) found that the results of GDS indicated developmental backwardness and imbalance in ASD children, and the DQ scores in fine motor ability, adaptability, language, and individual-social behavior were lower especially in the latter two functions compared with gross motor.

Wechsler Preschool and Primary Scale of Intelligence (WPPSI) (Watkins and Beaujean, 2014) was one of the three continuous intelligent scales created by Wechsler, a famous American medical psychologist, mainly for children with mental retardation aged 4–6.5 years. The WPPSI gave consideration to the ability of both language and operation, which may reflect the intelligence level of preschool and primary children.

Autism Diagnostic Observation Schedule (ADOS) (Randall *et al.* 2018) was the gold standard for diagnosis of ASD, identifying ASD symptoms of communication, interaction, game/imagination, stereotyped behaviors, and limited interests. According to the verbal expression level and age, the assessor should choose one of the four modules appropriately.

### Analytic approach

The results of the A/CIFR were compared between BL and PT to evaluate whether caregivers’ skills of family intervention improved and whether the longer daily family intervention period was beneficial. Second, by comparing assessment scales including S-M, GDS, WPPSI, and ADOS between BL and PT, children’s abilities and symptoms improved through family intervention. The influence of demographics, including the main caregiver, family structure, and household income, was also analyzed to determine whether the caregivers’ promotion of intervention skills was effective. SPSS 22.0 was used to analyze the data. The data included positive and negative values and were normally distributed (skew (< |2|); kurtosis (< |3.5|). A nonparametric test (Mann–Whitney *U* test or Wilcoxon signed-rank test) was used for comparisons between BL and PT for data from the scales previously mentioned. Spearman’s correlation was used to analyze the influence factors on caregivers’ skill promotion. *P* < 0.05 was considered statistically significant.

## Results

### Elementary analyses

#### Video quality

To ensure the video quality, the whole 120 videos (skew 1.00; kurtosis 4.42), 49 BL, and 71 PT videos were analyzed separately. The median length was 631 s and 617 s, respectively, and there was no difference between two groups (*P* > 0.05) over time.

#### Standardized measures

All 33 children completed the S-M before training. Two participants completed the WPPSI instead of the GDS because they were over four years old and diagnosed with intellectual disability by the initial clinician. Based on verbal expression level and age, different modules were used to evaluate 32 children before training. The last participant was diagnosed GDD so there was no ADOS assessment. Within six months after the training, standardized measures were lack of information because of dissatisfaction of regular follow-up evaluation on specialist clinic. The scores from S-M, GDS, WPPSI, and ADOS were 33–22, 31–25, 2–0, and 32–23 from BL to PT, respectively.

### Primary outcomes

Compared with BL, the mean score of Q1, Q3–Q10, and Q marked “×” was higher (*P* < 0.01, *P* < 0.05), with no change from BL to PT (*P* = 0.143) on Q2. Table 2 shows the performance on video-coded outcome measures by BL and PT.

**Table 2.** Comparisons of A/CIFR outcome on video data measures between baseline and post-training

Question	1	2	3	4	5	6	7	8	9	10	×	1–10
Baseline												
Mean (SD)	1.43 (1.06)	0.51 (1.00)	1.67 (0.97)	1.57 (1.10)	1.74 (1.11)	2.10 (1.23)	0.82 (0.81)	1.51 (1.26)	1.47 (1.06)	1.18 (1.07)	1.36 (1.12)	1.40 (0.89)
Post-Training												
Mean(SD)	2.68 (0.98)	0.79 (1.17)	2.76 (0.90)	2.55 (1.13)	2.96 (1.01)	3.17 (0.97)	1.61 (1.22)	2.30 (1.49)	2.73 (1.00)	2.58 (0.84)	2.29 (1.14)	2.41 (0.82)
R^2^	5.69	1.46	5.47	4.31	5.38	4.66	3.55	2.87	5.74	6.35	0.04	5.67
*P* value^a^	<0.01	0.143	<0.01	<0.01	<0.01	<0.01	<0.01	<0.01	<0.01	<0.01	<0.05	<0.01

a Significance at alpha (0.05/2) by Mann–Whitney *U* test.

According to the principle of self-contrast randomized paired design, 75 PT-BL pairs were analyzed. There was significant change from BL to PT (*P* < 0.01). Table 3 shows the contrast. Meanwhile, there was no correlation between the improvement of caregivers’ skills and duration of daily family intervention (ρ = −0.01, *P* > 0.05).

**Table 3.** Comparisons of A/CIFR outcome on 75-pair videos

Variable	Count	Mean (SD)	R^2^	*P* value^a^
Post-training-baseline	75	0.89 (0.11)	5.68	<0.01

a Significance at alpha (0.05/2) by Mann–Whitney *U* test.

### Secondary outcomes

Table 4 shows standardized measures outcomes between BL and PT. Compared with BL, in PT, children’s abilities and symptoms improved through family intervention, among which the adaptability of GDS, stereotyped behaviors, and the limited interests of ADOS were significant changes (*P* < 0.05, *P* < 0.01).

**Table 4.** Standardized measures outcomes between baseline and post-training

Variable Mean (SD)	S-M^a^	GDS^b^					ADOS^c^				
		Adaptability	Gross motor	Fine motor	Language	Individual-social behavior	Communication	Interaction	Communication, interaction (social affection)	Game/imagination	Stereotyped behaviors and limited interests.
Baseline	9.06 (0.70)	59.65 (16.59)	73.26 (14.02)	71.84 (17.82)	47.13 (14.54)	60.26 (11.62)	4.86 (2.00)	9.13 (2.93)	14.00 (4.20)	2.30 (1.30)	2.09 (1.42)
Post-Training	9.27 (0.77)	68.56 (14.30)	75.88 (20.33)	76.28 (14.56)	52.24 (18.25)	63.80 (14.86)	4.70 (2.18)	7.74 (3.53)	12.48 (5.20)	1.65 (1.43)	1.09 (1.08)
R^2^	1.11	2.18	0.37	1.15	1.01	0.77	−0.17	−1.20	−0.98	−1.85	−2.70
*P* value^d^	0.266	*P* < 0.05	0.711	0.248	0.314	0.443	0.863	0.229	0.329	0.064	*P* < 0.01

a S-M: baby-junior high school students ability of social life scale;

b GDS: Gesell Developmental Scales;

c ADOS: Autism Diagnostic Observation Schedule;

d significance at alpha (0.05/2) by Mann–Whitney *U* test.

### Correlation analyses

There was mild negative correlation between the rising mean scores of Q1–Q10 from BL to PT and parental education (ρ: −0.29, −0.29, *P* < 0.05), which suggested that higher parental education levels were not associated with improvement in caregivers’ intervention skills. There were no correlations between the improvement and other demographics, including the main caregiver, family structure, and annual household income. Table 5 presents the results.

**Table 5.** Correlation analysis between improvement and demographics from baseline to post-training

Variable		Father’s education	Mother’s education	Main caregiver	Family structure	Annual household income
Rising mean score	ρ	−0.29	−0.29	0.01	0.13	−0.19
	*P* value^a^	*P* < 0.05	*P* < 0.05	0.951	0.270	0.096

a Significance at alpha (0.05/2) by Spearman’s correlation.

## Discussion

First, caregivers can significantly improve their family intervention skills through short-term systematic training. Family-mediated intervention was proven as one of the 27 evidence-based methods by the American National Center for Autism Intervention and Professional Development in 2007 (Lord *et al.* 2018). At the same time, family-mediated intervention was defined as “parents apply personalized intervention strategies learned from systematic training to improve communication and social skills of children with ASD or reduce their various behavioral problems in the daily life.” At present, there is a lack of systematic and effective family-mediated intervention models in China (Ge and Dai, 2019). One probable reason is that caregivers are unable to master a series of intervention methods, including behavioral intervention, structured education, social intervention, sensory intervention, and comprehensive intervention. In their opinion, these interventions are for professionals, and they should be completed in hospitals, training institutions, or schools. The aim of family-mediated intervention models is to train caregivers to master and apply intervention strategies in home and play routines. The question is how long it will take to train them. On one hand, this study demonstrated that by only ten 90-minute lessons (total training time: 15 h), a two-week teaching cycle, a significant improvement in caregivers’ intervention skills was observed within six months, which suggests that caregivers can significantly improve their family intervention skills through short-term systematic training. On the other hand, this study did not find a correlation between the improvement and the length of intervention time, which suggests that there was no dose–effect relationship between them. It is possible that the theoretical knowledge of the CMI was relatively basic and simple for caregivers, so there was no need to spend a long time practicing. However, this also suggests that caregivers can master intervention skills. During a telephone follow-up with the parents of the participants, ninety-seven percent of participants took part in different intervention models, including family intervention, during a 6-month period, including training institutions, special education, and integrated education. Therefore, other factors may influence skill improvement and intervention duration, which should be studied further.

Second, CMI may contribute to the overall improvement of children’s abilities and remission of their symptoms. As core symptoms of ASD, social interaction and communication deficit were the focus of the training. It has been reported that a series of family intervention models mostly aimed to improve communication and social interaction skills of children with ASD actually contributed to their ability to share positive emotion, initiate communication, and social skills in different degrees (Kasari *et al.* 2010, Pajareya and Nopmaneejumruslers, 2012, Brian *et al.* 2017). However, another core symptom of ASD, stereotypical behaviors and limited interests, is rarely targeted in interventions. Previous research (Harrop *et al.* 2017) has demonstrated that family intervention effectively improved parents’ ability to respond to the symptoms instead of reducing it. This study found a comprehensive improvement in ability and the severity of the symptoms, among which the adaptability, stereotypical behaviors, and limited interests were especially significant. Nevertheless, as previously stated, other intervention models were used simultaneously, so the direct correlation between the overall improvement and CMI was difficult to evaluate. Meanwhile, comprehensive intervention was confirmed effective to some extent from another point of view.

Third, CIM can be applied in families with different demographics, especially ones with a lower basic education. Currently, the effect of the family intervention model on children with ASD is poorly understood. A systematic review (Shalev *et al.* 2019) analyzed the role of parent characteristics in parent-mediated interventions (PIM) for children with ASD. That review suggested that parent demographics (i.e., age, nationality, education, and family socioeconomic status), mental stress, and cognitive characteristics may influence the quality of family intervention regarding learning new strategies, goal-setting skills, and the ability to draw inferences about other cases from one instance, thus indirectly affecting intervention. Parental mental stress, as a hot research topic among those characteristics (Birkin *et al.* 2008), may have complex workings. For one thing, it could be suggested that parents with lower levels of baseline stress are more likely to attend PIM sessions, show higher engagement, and accept training from clinicians. It is also possible that parents with higher levels of baseline stress are more likely to need support and engage in PMI sessions. In the meantime, parental mental stress can be relieved by coaching to different degrees (Ingersoll and Wainer, 2013, Kuravackel *et al.* 2018). That review also found that maternal age and parent education were not related to the intervention effect. This study found that the lower the parental education was, the more significant the strategy application improvement was, although there was only with low correlation. The probable reason is that parents with a higher education are more likely to access to intervention strategies in daily learning and master some basic methods, resulting in less room for improvement and less obvious skills improvement, which implies CMI may be more suitable for families with a lower basic education. In addition, no correlation was found between caregiver outcomes and other demographics. Therefore, to conclude, CMI can be widely applied in families with different demographics.

Compared with various family intervention models overseas, there is a shortage in this research field in China. This study aims to compensate for the deficiency, providing a reference for clinical intervention for children with NDDs. At the same time, based on video-coded data from the A/CIFR scale, caregiver intervention skills are quantified, which contributes to quantitative assessment of caregivers’ behaviors and correlation analysis.

Of course, this study has certain limitations. First, the sample was relatively small, which led to gender difference in the samples compared with that of the prevalence of ASD. The primary outcome was to assess changes in parents’ intervention skills, and the secondary outcome was to observe changes in children’s abilities in a natural family environment, so the video recording could only be performed at home. Based on previous literature, only 10-minute video recording sessions were stipulated. Since many children with ASD exhibit social dysfunction, it is quite difficult to maintain effective interaction for 10 min. Additionally, as the Southwest Children’s Medical Center, nearly half the children who attend the hospital come from distant regions, so it is difficult for children to return to the hospital for follow-up after 3–6 months. Second, regarding the primary outcomes, the A/CIFR scale for video-coded data was subjective to some extent, although the author became familiar with the scale systematically and maintained quality control in the preliminary stage. Third, other interventions were not excluded, except CMI because of ethical issues, which may have some effect on the evaluation. The follow-up by telephone determined that the use of CMI strategies was relatively high frequency, which played an important role in the overall improvement for children. Additionally, the focus of CMI was the improvement of caregivers’ skills, which was mainly influenced by the intervention of CMI. Therefore, in future studies, clinicians should publicize the importance of follow-ups for NDDs – a disease that needs lifelong intervention. Meanwhile, more objective scales should be developed to quantify the intervention effect. What is more, RCTs are better for evaluation.

Training caregivers as mediators of a naturalistic, developmentally sensitive intervention allows for the integration of intervention into families’ daily activities, increasing access to intensive intervention within a child’s natural social context. CMI can significantly enhance the family-mediated intervention skills of caregivers in families with NDDs over a short period and help improve overall abilities and symptoms of children. As a family-mediated intervention model with few limitations, it can be applied to families with different demographic backgrounds. In conclusion, CMI can be considered an effective intervention measure, which deserves to popularized.
